# Fungal allies in saline soils: mineral mobilization, plant nutrient acquisition and salinity stress tolerance

**DOI:** 10.3389/fmicb.2026.1891929

**Published:** 2026-09-15

**Authors:** Himani Kaushik, Kunal, Rohit Kumar, Pankaj Kumar, Atin Kumar, Debasis Mitra, Mahendru Kumar Gautam, Caleb Ayomide Babatunde

**Affiliations:** 1 Department of Life Sciences, School of Allied Health Sciences, SGT University, Gurugram, India; 2 Faculty of Agricultural Sciences, GLA University, Mathura, India; 3 Department of Entomology, Sardar Vallabhbhai Patel University of Agriculture and Technology, Meerut, India; 4 School of Agriculture, Uttaranchal University, Dehradun, India; 5 Department of Microbiology, Graphic Era (Deemed to Be University), Dehradun, India; 6 School of Agricultural Sciences, Babu Banarasi Das University, Lucknow, India; 7 Pedology and Land Use Research Group, Department of Soil Resources and Environmental Management, Ekiti State University, Ado Ekiti, Nigeria

**Keywords:** salinity stress, beneficial fungi, mineral solubilization, nutrient uptake, plant-fungus interactions, sustainable agriculture

## Abstract

Soil salinity is an issue in agriculture. It also has a vast effect on decreasing agrarian productivity, as it limits the availability of nutrients and results in physiological stress on plants. Traditional methods of managing and treating soil cannot be used in saline and mineral-deficient conditions. As a solution to these problems, fungi are important biological agents for sustainable solutions. This review concentrates on fungal diversity and its aptitude to alleviate salinity stress and enhance nutrient availability through mineral solubilization and mobilization and, in selected plant–fungus associations, facilitate subsequent nutrient acquisition by the plant. The adverse effects of salinity on plant growth, nutrient uptake, and overall physiological performance are discussed, and the mechanisms of stress mitigation and enhancement of mineral availability mediated by fungi are described, such as organic acid synthesis and ion homeostasis. Plant-fungal interactions enhance salinity stress resistance, as discussed in this review. Moreover, developments in the field of molecular and omics-based studies cannot be overestimated in terms of acquiring an understanding of the genetic and molecular pathways of plant-fungal interactions in saline and mineral-deficient environments.

## 1 Introduction

Salinity is one of the most important limitations for sustainable crop production, which impacts plants by osmotic and ionic stress, and by mineral nutrition. The excessive accumulation of Na^+^ and Cl^–^ affects the availability, uptake, transport and cellular ion homeostasis, which ultimately affects plant growth and productivity (Porcel et al., 2012; Bhupenchandra et al., 2024). These effects can be mitigated by beneficial fungi, such as arbuscular mycorrhizal fungi (AMF), plant growth-promoting fungi, endophytes and mineral solubilizing fungi, which can enhance nutrient mobilization and acquisition, maintain a favorable K^+^/Na^+^ balance, facilitate osmotic adjustment and phytohormonal regulation, and strengthen antioxidant defense. Fungal-mediated mineral mobilization and salinity tolerance, therefore, are closely linked processes that can be involved in plant adaptation in salt-affected soils.

The widespread occurrence of soil salinization around the world also highlights the significance of these biological solutions. Salinity impacts 10.7% (1,381 million ha) of the land area and almost 10% of cropland worldwide (FAO, 2024). Its distribution, however, is quite differential between regions (Table 1), where 42.1% of the land is under salt-affected soils in the Pacific, 17.7% in the Near America and North Africa and 11.7% in Latin America and the Caribbean. Asia has a relatively small percentage (7.4%), but high levels of intensive cultivation and irrigation make salinity more relevant in agriculture in this region. Additionally, temperature, evaporation, precipitation, irrigation requirements and seawater intrusion changes due to climate change could exacerbate soil salinization especially in vulnerable areas (FAO, 2024). The trends suggest the importance of sustainable approaches to improve nutrient availability and plant salinity tolerance, such as beneficial fungal interventions.

**TABLE 1:** Areas of salt-affected soils at the regional level.

Region	Salt-affected area (km^2^)	Total land area (km^2^)	% of land affected
Pacific	3,570,000	8,472,605	42.1%
Europe and Eurasia	2,378,209	27,049,956	8.8%
Latin America and the Caribbean	2,352,857	20,026,933	11.7%
Near America and North Africa	2,303,461	13,033,953	17.7%
Asia	1,543,269	20,722,790	7.4%
Africa	883,795	22,046,043	4.0%
North America	779,912	17,936,120	4.3%
Total	13,811,503	129,288,400	10.7%

Salinity-induced nutritional imbalance is a significant constraint on plant growth because salt stress also affects water uptake, nutrient uptake, and ion homeostasis in the cell. Although plants require essential macro and micronutrients for metabolic, photosynthetic, enzymatic, developmental and stress responses, most of the soil nutrient pool is in sparingly soluble mineral forms and is therefore poorly available for plant uptake (Khuna et al., 2021). In addition, salinity affects nutrient availability by altering soil water potential, rhizosphere chemistry, and nutrient mobility. In particular, the uptake of K^+^ may be affected by excess Na^+^, which also disrupts the cellular K^+^/Na^+^ balance, and the acquisition of P, Ca, Mg, Fe, Zn and other nutrients may also be disturbed (Porcel et al., 2012). Therefore, mineral nutrition disturbance is a part of the plant’s response to salinity, not just a result of growth reduction.

These nutritional limitations can be overcome by beneficial fungi through interrelated mechanisms of mineral mobilization and stress regulation. Mineral-solubilizing fungi produce protons, organic acids and extracellular enzymes to aid in the acidification, chelation, dissolution and mobilization of minerals that are not otherwise accessible. In addition, mycorrhizal fungi have a very large extraradical hyphal network that searches soil outside the root zone, i.e., nutrient depletion zone (Boorboori and Lackóová, 2025). These fungal associations are especially beneficial in saline situations where root growth and nutrient movement are limited. Better nutrient acquisition (P, K, Ca, micronutrients) and decreased accumulation of Na^+^ can help maintain a favorable ionic balance and nutritional status of plants.

Fungal-mediated salinity tolerance is not just about nutrient mobilization. Beneficial fungi may affect root architecture and water relations, modulate osmotic adjustment, regulate phytohormonal signaling, enhance antioxidant defense and modulate ion transport and compartmentation (Abdallah et al., 2026). The combined effect of increased antioxidant enzyme activities and regulation of transport processes associated with Na^+^ exclusion, K^+^ retention, ion sequestration and water movement all help to decrease ionic, osmotic, and oxidative damage (Wang et al., 2024; Boyno et al., 2025). In conclusion, salinity tolerance is a multi-faceted phenomenon that involves soil chemical, nutritional, physiological, biochemical and molecular processes, which are mediated by fungi.

Although there are growing indications of these positive impacts, there is still a lack of synthesis of existing knowledge. The studies on mineral-solubilizing fungi are mainly related to the mobilization of nutrients and biofertilization, while the studies on the role of fungi in salinity are mainly related to the antioxidant response, osmotic regulation and ion homeostasis. Furthermore, endophytic, rhizospheric and free-living beneficial fungi have received comparatively less attention than AMF. Thus, the interconnection among the functional aspects of fungal mineral solubilization, nutrient uptake, ion homeostasis, and salinity tolerance in plants remains poorly understood, especially at the molecular and omics levels. The results of the laboratory tests are further complicated by variation among fungal taxa, host plants, soil properties, nutrient availability, and salinity intensity.

A major limitation in the available literature is the lack of balanced experimental levels. Laboratory studies offer detailed information on fungal functional traits but are typically conducted under simplified conditions; greenhouse studies offer a greater insight of plant–fungus interactions under controlled salinity. Field studies offer the greatest ecological relevance but are less common and often have more variability in establishment of fungi and responses of plants. Therefore, evidence of fungal ability to solubilize minerals and tolerate salinity should be considered as a continuum of experiments and not a single entity. The ability to distinguish between the efficacy of the fungus in the controlled environment compared to the ability to reproduce efficacy under field conditions is therefore critical when assessing the practical potential of fungal inoculants.

Therefore, this review brings together current knowledge of beneficial fungi at the mineral nutrition-salinity tolerance interface, including mycorrhizal, endophytic, rhizospheric and other mineral-solubilizing fungi. Special focus is given to mechanisms of nutrient mobilization, ionic and osmotic homeostasis, antioxidant regulation, plant physiology and adaptation, and novel molecular and omics-based knowledge. The review focuses on key knowledge gaps and future research priorities for the development of fungal inoculants, microbial consortia, and microbiome-based strategies for sustainable crop production in salt-affected soils, integrating these processes within a plant–fungus–soil framework.

### 1.1 Review methodology

This article presents a narrative review based on a structured literature search and study-selection procedure to synthesize current knowledge on beneficial fungal–plant interactions in mineral mobilization, nutrient acquisition and salinity tolerance. Given the diversity of fungal groups, host plants, and experimental systems, the review integrates and critically discusses evidence from laboratory, greenhouse and field studies to identify key mechanisms, knowledge gaps and future research priorities. The most relevant peer-reviewed research and review articles were retrieved from leading scientific databases, such as Web of Science, ScienceDirect, PubMed, and Google Scholar. The literature review encompassed articles majorly published between 2010 and 2025 due to the dynamic growth of studies in this area. Relevant studies were identified using a combination of the following keywords: beneficial fungi, plant-fungus interaction, mineral solubilization, mineral solubilization, mineral mobilization, arbuscular mycorrhizal fungi, endophytic fungi, rhizospheric fungi, salinity stress, salt-tolerant, salinity tolerance, nutrient uptake, fungal metabolites, plant growth promotion, and crop productivity.

The retrieved articles were screened using their titles and abstracts before full text was assessed for eligibility according to the predetermined inclusion/exclusion criteria. Only studies pertaining to beneficial fungi, mineral mobilization, nutrient acquisition, salinity responses and related physiological, biochemical or molecular mechanisms were included and those that were not relevant or sufficiently documented were excluded. Foundational studies were also identified by using reference lists. The selected literature was thematically synthesized, focusing on fungal diversity, mobilization of nutrients, plant nutrient acquisition, ion and osmotic regulation, antioxidant and hormonal regulation, and evidence across experimental systems. Given the wide variety of studies in the literature, a qualitative and thematic synthesis was undertaken without meta-analysis.

#### 1.1.1 Inclusion criteria

(i) Studies on plant-beneficial fungi in soil, roots, or the rhizosphere.
(ii) Studies involving AMF, endophytic fungi, rhizospheric fungi, plant growth-promoting fungi, mineral-solubilizing fungi, or other functionally relevant beneficial fungal groups.
(iii) Studies examining fungal-mediated mineral solubilization, mobilization, acquisition, or improved availability of macro and micronutrients.
(iv) Studies evaluating effects of fungi on plant responses to salinity or salt-associated nutrient stress.
(v) Studies on physiological, biochemical, morphological, nutritional, molecular, genomic, transcriptomic, proteomic, metabolomic, or other omics-related responses associated with fungal-mediated stress tolerance.
(vi) Studies involving laboratory, greenhouse, pot, hydroponic, field, or other experimentally relevant plant–fungus systems.
(vii) Studies published before and after the 2010–2025 period that provided foundational evidence or established mechanisms directly relevant to the scope of the present review.

#### 1.1.2 Exclusion criteria

(i) Publications that are not related to beneficial fungal interactions with plants, soil nutrient availability, mineral solubilization, or salinity stress.
(ii) Studies dealing with pathogenic fungi or fungal diseases without having any connection to plant nutrition or salinity tolerance.
(iii) Studies on fungi without a plant-associated, soil-related, mineral-solubilizing, or salinity-stress component.
(iv) Publications that lack sufficient information on the fungal taxon, experimental treatment, plant response, or mechanism under investigation.
(v) Studies focussed on salinity issues from a physicochemical perspective without examining plant–fungus interactions.
(vi) Non-peer-reviewed literature, such as news articles, non-scientific web content, and publications lacking adequate scientific information.

### 1.2 Causes of soil salinity and mineral unavailability

Salinity affects the physicochemical and biological characteristics of soil such as pH, osmotic potential, mineral equilibria, water availability, soil structure and microbial activity, which affect the solubility of soil nutrients and plant uptake (Bidalia et al., 2019). Soil pH is also a significant factor affecting nutrient availability (Alam et al., 1999), but pH is not the only factor in limiting nutrient availability in saline soils. For instance, phosphorus (P) availability decreases in acidic soils due to its sorption by Fe and Al oxides and hydroxides (Fahmi et al., 2023), whereas under alkaline conditions, its precipitation with Ca^2 +^ and Mg^2 +^ forms poorly soluble compounds (Elbasiouny et al., 2020). Salinity also affects mineral equilibria and the mobility of nutrients, and deterioration of soil structure due to Na^+^ (especially under sodic conditions) can also limit water movement and nutrient transport to the roots (Ehtaiwesh, 2022; Husson, 2023).

Most significantly, salinity affects the ability to access nutrients due to osmotic stress and ion competition. High salt concentrations reduce the availability of soil water to roots, which decreases water uptake and the delivery of dissolved nutrients to the root surface. Excess Na^+^ also raises Na^+^/K^+^ and Na^+^/Ca^2 +^ ratios and interferes with the uptake and transport of essential cations. This decreases the uptake of K^+^, Ca^2 +^ and Mg^2 +^, which impacts membrane stability, enzyme activity and cellular ion homeostasis, thereby reducing nutrient-use efficiency and plant growth (Ahmed et al., 2024; Patil et al., 2026). Therefore, under salinity, nutrient deficiency is due to reduced nutrient mobility, impaired root function, and competitive inhibition of nutrient uptake.

There are two sources of soil salinity, namely, natural (primary) and anthropogenic (secondary) (Singh, 2021). Natural salinization can be caused by weathering of the parent material, geological salt deposits, fluvial and marine salt inputs, while inappropriate irrigation, poor drainage, groundwater exploitation and intensive agricultural practices are typical causes of secondary salinization. High evapotranspiration and low rainfall rates are also likely to lead to salt accumulation in arid and semi-arid regions, and seawater intrusion, sea spray, storms and cyclones are also likely to affect coastal soils (Kumar et al., 2020). These processes result in an unfavorable rhizosphere environment including osmotic stress, ion toxicity, nutrient imbalance, and a changed plant–microbe interaction (Bidalia et al., 2019).

Salinity also alters the rhizosphere microbiome and has significant implications for nutrient cycling. High salt levels can reduce the growth and activity of microorganisms and shift the microbial community toward more salt-tolerant groups. These alterations can result in a decrease in biological processes such as nutrient mineralization, fixation, solubilization and mobilization, including P, K and Zn availability (Yadav et al., 2022). Therefore, with saline conditions, the nutrient availability and soil fertility may be further reduced due to decreased microbial activity (Haj-Amor et al., 2022).

In this context, beneficial fungi play a significant role as a biological connection between soil nutrient pools and plant acquisition. Salinity can have negative impacts on fungal growth, colonization and community structure, however, salt-tolerant beneficial fungi, such as arbuscular mycorrhizal fungi (AMF) can at least offset the nutrient limitation imposed by salinity. AMF also play a significant role in ionic homeostasis by preventing excess accumulation of Na^+^ and enhancing the absorption of K^+^, Ca^2 +^, and other nutrients, thus regulating K^+^/Na^+^ balance (Ahmed et al., 2025; Gupta et al., 2021). Thus, beneficial fungi may help to mitigate the multifaceted responses to osmotic stress, ionic imbalance and nutrient deficiency, suggesting their use as a viable biological approach to enhancing nutrient uptake and plant productivity under saline conditions.

### 1.3 Mechanistic distinction between mineral solubilization, mobilization and plant nutrient uptake

The processes of mineral solubilization, mobilization and plant nutrient uptake are separable but interrelated. Mineral solubilization is the mineral dissolution process of poorly soluble mineral phases by mechanisms like proton mediated acidification, ligand exchange and chelation. Depending on the mineralogy, soil pH and rhizosphere conditions, fungal organic acids might also help mobilize mineral-associated nutrients.

Mineral mobilization is an umbrella term that refers to the release, desorption, redistribution or conversion of nutrients from relatively inaccessible pools of nutrients in the soil, to forms or locations that may be accessible to plants or microorganisms. Thus, mobilization of nutrients can occur through more than one mechanism including solubilization, and may also occur through fungal exploration of the hyphae and enzymatic mineralization of organic nutrient pools.

Then plant nutrient uptake takes place by acquisition of available nutrients by roots or in the case of mycorrhizal association by acquisition by fungal tissue and transfer through the symbiotic interface. The higher the concentration of soluble nutrients, the more available or mobilized nutrients are present, while the higher the concentration of nutrients in plant tissues or nutrient flux quantified, the better evidence of enhanced plant nutrient acquisition will be. Extraradical hyphae are able to search soils outside of the root depletion zone and can mediate nutrient uptake and transport, especially for phosphorus, without necessarily directly solubilizing minerals in the AMF associations.

Mineralization is different from solubilization which is the biological breakdown of organic nutrient compounds to inorganic compounds (e.g., fungal phosphatase or phytase hydrolysis of organic phosphorus). These differences explain the importance and involvement of fungal organic acids, siderophores, plant-available extracellular enzymes and the AMF plant nutrient transfer in plant nutrition.

### 1.4 Microbes as eco-friendly alternatives to chemical soil management

In conventional farming, high crop productivity is obtained by using chemical fertilizers, pesticides, intensive tillage, and other energy-intensive inputs (Lal, 2020). Chemical fertilizers provide the essential nutrients quickly, but when overapplied, they can lead to nutrient buildup, soil salinization, soil degradation, and the loss of indigenous soil microbial communities (Anwar et al., 2021; Hou et al., 2025). Likewise, conventional breeding and biotechnological approaches for developing salt-tolerant crops are often time- and resource-intensive (Latif et al., 2023). The constraints underscore the need to develop environmentally-friendly strategies that can help to increase nutrient availability and increase plant salinity tolerance.

Soil health is a prerequisite for crop productivity especially in nutrient-deficient soils, soil degradation, pH and porosity changes, salinity and nutrient toxicity (Shahane and Shivay, 2021). Biological soil management is a potential solution to the excessive use of agrochemicals (Hossain and Sultana, 2020). Fungi are beneficial soil microorganisms due to their large hyphal network, versatility in metabolic activities, and high interaction with plant roots. Under nutrient-limiting and saline conditions, AMF, *Trichoderma*, *Penicillium* and *Aspergillus* can enhance nutrient availability and plant performance (Abbas et al., 2019; Elgharably and Nafady, 2021).

Mineral-solubilizing fungi use various strategies to make nutrients available to plants when they are not readily available. The mineral-associated metals can be mobilized by rhizosphere acidification and production of organic acids by *Penicillium* and *Aspergillus* spp. and by chelation by siderophores. Other extracellular enzymes produced by fungi, such as phosphatases and phytases, also play a role in the mineralization of organic nutrient pools (Kaur et al., 2021). *Trichoderma* spp. also has the ability to facilitate nutrient acquisition by mineral solubilization, production of extracellular enzymes and root growth modification. AMF use a complementary mechanism by forming extraradical hyphae, which are in contact with soil beyond the zone of root nutrient depletion, which means that they explore more soil and can acquire and transport relatively immobile nutrients such as phosphorus. Therefore, under abiotic stress, the AMF associations improve plant growth, nutrient uptake, water relations and aboveground productivity (Phour and Sindhu, 2024).

Under salinity, osmotic stress, ion toxicity and nutrient imbalance limit plant growth, making these fungal functions especially relevant. AMF and other beneficial fungi can help overcome these limitations by enhancing nutrient and water uptake, maintaining Na^+^/K^+^ balance, modulating stress-related pathways, and boosting antioxidant defense mechanisms (Boyno et al., 2025). Therefore, the ability to tolerate salinity and fungal-mediated mineral solubilization is functionally linked because better nutrient availability and ion homeostasis can contribute to plant growth under saline conditions.

The field performance of fungal bioinoculants is variable. They depend on the type of fungus, the host genotype, soil properties, salinity, climatic conditions, interaction with indigenous microorganisms, formulation of the inoculum and its application. Other limitations for widespread use include problems with mass production, storage stability, shelf life, and quality control, especially for obligate biotrophic AMF. Thus, fungal inoculants should be seen as part of an integrated soil and nutrient management system, not as a substitute for mineral fertilizers.

The review emphasizes the diversity, functional properties and molecular and genomic aspects of the mineral solubilization capacity of fungi, especially AMF, *Trichoderma*, *Penicillium* and *Aspergillus*. It also explores the role of fungal processes including production of organic acids, siderophores, extracellular enzymatic activity and hyphal nutrient transport in plant nutrition and salinity tolerance. The interconnections of these processes are essential for the formulation of effective fungal bioinoculants for nutrient-efficient, salt-resilient and sustainable agricultural systems.

## 2 Diversity of fungi in saline soil and mineral-deficient soil

Fungi play a vital role in soil ecosystems in terms of nutrient cycling, decomposition of organic matter, soil aggregation, and productivity of plants (Chaurasia and Bharati, 2021). Their diversity is especially relevant in saline and mineral poor soils, where osmotic stress, ion toxicity, nutrient availability and low water potential are strong environmental filters that influence fungal communities (Yang and Sun, 2020). As a result, fungi that survive in these environments are frequently not just stress tolerant, but actually functionally adapted to stress.

The ability of halotolerant fungi to tolerate salinity involves coordinated physiological and molecular mechanisms such as the accumulation of compatible osmolytes, maintenance of ion homeostasis, modification of membrane properties, antioxidant defense, and activation of stress-responsive signaling pathways. The high-osmolarity glycerol (HOG) pathway is especially significant in fungal osmoadaptation as it controls glycerol accumulation and helps to maintain cellular water balance. Under conditions which inhibit the ability of less adapted microbial groups to carry on metabolic activity and ecological function, these adaptations enable salt-tolerant fungi to continue these activities.

Fungi are found in a variety of ecological roles including saprotrophs, endophytes, mycorrhizal symbionts, mutualists, predators, and pathogens (Akter et al., 2025). Plant-associated fungi such as endophytic, mycorrhizal, phyllosphere, and rhizospheric fungi include a variety of plant growth-promoting fungi (PGPF) that are beneficial to plants in terms of nutrition and stress resistance (Abo Nouh et al., 2021; Akter et al., 2025). Their importance increases when salinity is combined with mineral deficiency as high levels of salinity can also limit nutrient availability and root uptake.

Fungi under stress can promote plant growth by releasing nutrients and by altering plant physiology. Fungi can increase the availability of poorly accessible nutrients through several mechanisms, including inorganic mineral dissolution, organic nutrient mineralization, ligand-mediated mobilization and hyphal exploration of nutrient-depleted soil. Fungi with extensive hyphae can also penetrate soil outside the nutrient-depleted root zone and fungi can also produce the plant hormone indole-3-acetic acid (IAA) which can alter root architecture and enhance uptake of nutrients (Bhupenchandra et al., 2024). Therefore, the benefits of fungi in nutrient deficient soils are due to both biochemical mineralization and better root-soil interactions.

*Aspergillus*, *Penicillium*, and *Trichoderma* are commonly found genera that are known for their ability to solubilize nutrients and alleviate plant stress (Chandra et al., 2020). They are effective in relation to their metabolic versatility, fast rhizosphere colonization, production of extracellular enzymes and excretion of mineral-solubilizing metabolites. In particular, *Aspergillus* and *Penicillium* are good producers of organic acids that can mobilize insoluble mineral nutrients, while *Trichoderma* is characterized by its nutrient mobilization ability and its high rhizosphere competence and antagonism against phytopathogens. In addition, *Trichoderma* spp. can stimulate root growth, antioxidant defense and plant performance under salinity, which makes it a suitable bioinoculant in saline agriculture (Ding et al., 2021).

Other mechanisms of salinity tolerance by fungi include osmotic adjustment and oxidative stress control. For instance, *Penicillium chrysogenum* can be used to increase the production of proline and soluble sugars and to improve antioxidant defense, thus mitigating the oxidative damage caused by salt stress (Dargiri et al., 2025). Likewise, *Aspergillus ochraceus* is able to produce IAA and boost the levels of antioxidant enzymes (POD, SOD, and PPO) and decrease H_2_O_2_ accumulation (Badawy et al., 2021). These observations suggest that the role of these fungi is not limited to nutrient solubilization, but also includes the regulation of plant redox balance, osmotic homeostasis, and stress-responsive pathways.

Another mechanism of adaptation to saline and nutrient poor soils is the mycorrhizal fungi. AMF enhance the uptake surface of the roots and help in the uptake of relatively immobile nutrients, such as P and Zn. AMF can also enhance K^+^/Na^+^ homeostasis, water relations, antioxidant defense and photosynthetic performance under salinity (Ahmed et al., 2025). These responses include regulation of membrane ion transport, aquaporins, osmotic adjustment, and stress-related signaling, and indicate that mycorrhizal-mediated salinity tolerance is not only in terms of enhanced nutrient uptake.

Other fungi, such as salt-adapted ectomycorrhizal fungi (ECMF) and dark septate endophytes (DSEs), are also significant members of the fungal communities in saline and nutrient-poor soils. DSEs are melanized septate hyphae and are often associated with plants under stress conditions and may affect root growth, antioxidant activity, osmotic regulation and ion homeostasis. Recent studies show that salt-adapted ECMF and DSEs may reduce Na^+^ accumulation, Na^+^/K^+^ ratio and increase root growth and antioxidant defense, and mixed inoculation often yields better results than any of the fungal inoculants alone (Ding et al., 2026). Responses to the DSE, however, are highly strain, host and stress dependent and therefore cannot be assumed to be beneficial when associated with plants.

Together, the ability of fungi to adapt to saline and mineral deficient soils is a complex interplay of fungal stress resistance, nutrient assimilation, root colonization, and fungal modulation of host physiology. However, most of the evidence available is based on single isolates in controlled conditions. Furthermore, there are species of genera with opposing ecological roles, and taxonomic abundance does not necessarily translate into beneficial activity, as in the case of genera like *Aspergillus*, *Penicillium*, *Fusarium* and *Trichoderma*. Future research should combine community profiling with functional, transcriptomic and metabolomic technologies to discover locally adapted fungal strains and compatible consortia that are consistently able to enhance plant performance in naturally saline and nutrient-limited soils.

## 3 Impact of salt stress and nutrient deficiency on plants

The importance of mineral nutrition on plant growth is that nutrients control photosynthesis, enzyme activity, protein synthesis, energy metabolism and osmotic adjustment. K, Zn and P are involved in a number of metabolic pathways related to plant growth and stress response (Xue et al., 2022). Deficiency thus may result in chlorosis, limited root and shoot growth, decreased photosynthesis, delayed reproductive development, and decreased productivity (Table 2). For instance, P deficiency slows down flowering and delays root growth, while Fe deficiency causes interveinal chlorosis, a characteristic symptom (Pandey et al., 2024). The nutritional disorders can be exacerbated when salinity conditions are present, as salinity affects nutrient availability, uptake, transport and utilization.

**TABLE 2:** Effects of mineral deficiencies on crop plants.

S. no.	Minerals	Effects of mineral deficiency on crop plants	Plant	References
1.	Boron (B)	Reduced protein and photosynthesis, decreased chlorophyll, changed mineral uptake (Mg, P and Zn), enhanced antioxidant activity, impaired reproductive development, and leaf chlorosis.	Watermelon	Moustafa-Farag et al., 2016
2.	Calcium (Ca)	Tip burn, necrosis of young leaves, root tip death, and reduced yield.	Groundnut, celery	Singh and Dadhe, 2022
3.	Copper (Cu)	Chlorosis of young leaves, stunted growth, stem melanosis, curling of leaf margins, apical meristem damage, reduced fruit formation, impaired water transport, affected pollen, seed viability, and embryo development.	Maize, citrus, wheat	Burkhead et al., 2009; Singh and Dadhe, 2022
4.	Iron (Fe)	Causes interveinal chlorosis, with younger leaves turning white (complete chlorosis), and reduced photosynthesis.	Soybean, grapevine	Santos et al., 2015; Rustioni et al., 2018
5.	Magnesium (Mg)	Interveinal chlorosis on older leaves, reddening leaves, poor ribosome activity, slows chlorophyll production, and photosynthesis.	Tobacco, cotton, Soybean	Atabayeva et al., 2020; Singh and Dadhe, 2022
6.	Manganese (Mn)	Photosystem II stability and functionality are affected by interveinal chlorosis, necrotic patches, and gray specks; a latent deficiency without obvious symptoms limits crop yield in Mn-poor areas.	Wheat, barley, oat	Schmidt et al., 2016; Singh and Dadhe, 2022
7.	Nickel (Ni)	Chlorosis on young leaves, initiating from the base, gradually spreads downward to the leaves.	Potato	Shukla and Gopal, 2009
8.	Nitrogen (N)	Lower crop yield, reduced vegetative development, yellowing of the leaves, poor photosynthesis, and nutritional imbalance.	Sweet orange, pummelo	Huang et al., 2021
9.	Phosphorus (P)	Reduced root and shoot growth, lower leaf number, impaired photosynthesis, carbohydrate accumulation, and increased oxidative stress.	Sour pummelo	Meng et al., 2021
10.	Potassium (K)	Leaf margin chlorosis, reduced root and shoot growth, inhibited enzyme and nitrogen metabolism, decreased root activity, impaired nutrient uptake, and increased osmotic stress.	Maize	Du et al., 2017
11.	Sulfur (S)	Chlorosis of young leaves, stunted growth, and symptoms of nitrogen deficiency.	Tea, crucifers	Singh and Dadhe, 2022
12.	Zinc (Zn)	Reduced photosynthesis, altered chloroplast pigments, short internodes, smaller leaves, white banding at the base of leaves, delayed maturity, and lower crop production.	Mungbean	Samreen et al., 2017

Plants are sensitive to salinity due to interrelated osmotic stress, ionic toxicity, oxidative stress, inhibition of photosynthesis, hormonal imbalance and mineral nutrition disturbance. First, reduced water uptake by the roots due to reduced soil water potential leads to physiological water deficit and limits cell expansion. Upon long exposure, Na^+^ and Cl^–^ ions are deposited in the plant tissue, which disrupts the ion homeostasis in the cell (Parihar et al., 2015; Singh, 2021; Choudhary et al., 2023). Excess Na^+^ is especially harmful because it competes with K^+^ and disrupts K^+^-dependent functions like stomatal regulation, protein synthesis and osmotic adjustment.

Osmotic and ionic disturbances also contribute to increased production of reactive oxygen species (ROS). If the production of ROS is higher than the antioxidant capacity of plant cells, oxidative damage to membranes, proteins, pigments and nucleic acids will happen (Arif et al., 2020). Salinity also inhibits photosynthesis by causing stomatal closure, limiting CO_2_ availability, breaking down chlorophyll, disrupting photosynthetic electron transport and inhibiting carbon fixation. All these effects together lead to chlorosis, necrosis, growth inhibition, early senescence and wilting (Das et al., 2024). Modifications in hormonal signaling, such as abscisic acid and growth-regulating hormones, in response to salt stress further alter stomatal behavior, root architecture, shoot development and stress adaptation.

Mineral nutrient deficiencies are an important consequence of salinity. NaCl at high concentrations disrupts nutrient uptake through ionic competition, inhibition of root growth, changes in membrane selectivity, changes in rhizosphere chemistry, and by limiting transpiration-driven transport. Na^+^–K^+^ antagonism is especially significant because when cellular K^+^ is lost, osmotic regulation and enzyme activity are impaired. Salinity can also interfere with Ca^2+^ homeostasis and limit nutrient uptake or use for P, N, Fe, Zn, Mg, Mn and others. Therefore, some symptoms observed in salt-stressed plants may be due to secondary nutritional imbalance and ionic toxicity.

Salinity and nutrient deficiency may thus interact to produce a reinforcing stress response. Salinity is known to limit nutrient uptake, but plant mineral nutrition deficiencies decrease their ability to perform photosynthesis, antioxidant defense, membrane stability, osmotic adjustment and metabolic activity, thus increasing their susceptibility to salt stress. Lack of Mg, Fe, Mn and N further reduces photosynthesis, while lack of K and Ca reduces ion homeostasis and membrane function. Likewise, the limited P availability reduces energy metabolism and root development, thereby reducing nutrient uptake in saline conditions. Table 3 presents the nutritional restrictions caused by salinity and their potential fungal-mediated mitigation.

**TABLE 3:** Nutritional constraints imposed by salinity and their potential fungal-mediated mitigation.

Nutrient/ion	Salinity-associated constraint	Consequence for plants	Potential fungal contribution
P	Reduced availability through precipitation/sorption and altered rhizosphere chemistry	Reduced root development and energy metabolism	Organic-acid-mediated solubilization; phosphatases/phytases; AMF-mediated acquisition
K^+^	Competition with Na^+^ and impaired uptake	Disturbed osmotic regulation and enzyme activity	Improved K^+^ acquisition and maintenance of K^+^/Na^+^ balance
Ca^2 +^	Competition/altered availability under saline conditions	Membrane and signaling disturbances	Improved Ca^2 +^ acquisition and root function
Fe^2+^	Reduced availability under altered soil chemistry	Chlorosis and impaired photosynthesis	Siderophore-mediated Fe mobilization
Zn^2+^	Reduced availability and altered transport	Enzyme and metabolic dysfunction	Fungal mobilization and enhanced acquisition
Mg^2 +^	Altered uptake under salinity	Impaired chlorophyll formation and photosynthesis	Improved mineral acquisition through fungal–root interactions

In total, the productivity of crops decreases under combined salinity and nutrient stress due to reduced water uptake, ion toxicity, increased ROS levels, decreased photosynthesis, hormonal imbalance and limited nutrient uptake. The net result of these processes is a restriction of biomass production, reproductive growth, and yield. Hence, nutritional imbalance must be regarded as a part of salinity stress and not as a separate stress. These interconnected effects are summarized in Figure 1, and the possible contribution of PGPF to improve nutrient uptake, ion homeostasis, antioxidant defense and plant growth under combined salinity and nutrient stress is highlighted.

**FIGURE 1:**
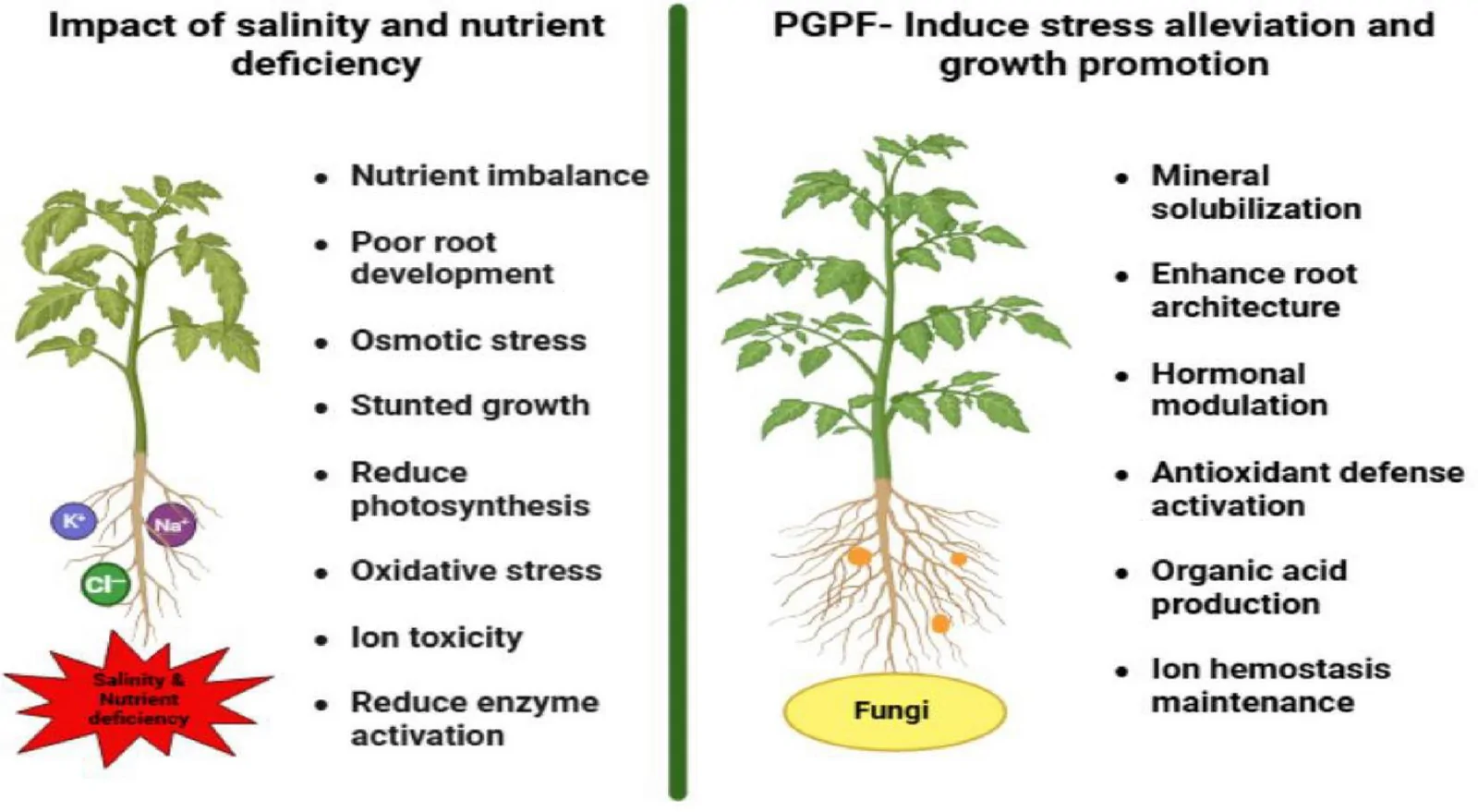
Mechanisms of fungal-mediated alleviation of salinity and nutrient stress in plants.

## 4 Role of fungi in salt stress mitigation and mineral uptake

### 4.1 Mechanisms of fungal-mediated salinity stress mitigation and mineral mobilization

The beneficial fungi reduce salinity stress in a variety of interconnected ways including mineral mobilization, selective nutrient uptake, ion homeostasis, osmotic adjustment, antioxidant defense, hormonal regulation and modification of root architecture (Evelin et al., 2019; Gupta et al., 2021; Aizaz et al., 2023). These processes are not independent, but synergistic, and they all help to enhance plant nutrition and physiological stability in saline environments.

#### 4.1.1 Nutrient acquisition and mineral mobilization

Fungal-mediated nutrient acquisition under salinity is not just limited to P but also other essential elements such as K, Fe, Zn, and Ca. The extraradical hyphae of AMF are responsible for enhancing P acquisition, while endophytes and phosphate-solubilizing fungi mobilize insoluble P and can also aid in N nutrition (Abo Nouh et al., 2021; Gupta et al., 2021). Salinity is especially important with respect to improved K^+^ uptake since K^+^ competes with Na^+^ and is important to maintain a favorable K^+^/Na^+^ ratio. Enzymes, photosynthesis, membrane integrity and stress signaling can be further enhanced by fungal mobilization of Zn, Fe and Ca. Therefore, fungal mineral solubilization should not be viewed as a sole mechanism for P acquisition but as a part of a larger mechanism to restore nutrient and ionic balance.

Fungal solubilization of inorganic minerals can be primarily considered as a chemical process resulting in increased dissolution of sparingly soluble mineral phases. Organic acids can act as proton donors, acidify the microenvironment, chelate mineral-associated cations and engage in ligand exchange at mineral surfaces. The influence of these mechanisms varies with the mineral phase and environmental factors. In the case of Ca^2+^-phosphate system, proton-mediated dissolution and complexation of Ca^2 +^ may promote phosphate release, while Fe^2+^ and Al^3+^ associated phosphates may be more sensitive to the complexation of metals and organic acid-mediated ligand exchange. Therefore, the capability of an isolate in laboratory conditions to produce organic acids need not be considered as a general indicator of its capacity in field conditions, as soil buffering, mineralogy and persistence of acids have a significant impact on results (Veresoglou et al., 2011; De Oliveira Mendes et al., 2014; Yadav et al., 2015; Din et al., 2020; Devi et al., 2022). *Aspergillus tubingensis*, for example, has been reported to release mineral-associated elements through the production of organic acids, and the role of specific organic acids in releasing these elements is dependent on the mineral substrate and experimental conditions (Yadav et al., 2015; Din et al., 2020).

Fungal phytases and phosphatases facilitate mineralization process by releasing P from organic molecules. Phytase activity has been reported in *Aspergillus chiangmaiensis*, *A. pseudopiperis* and *A. pseudotubingensis* (Khuna et al., 2021), and phosphatase activity in fungi like *Rhizophagus irregularis* and *Talaromyces helicus* (Della Mónica et al., 2020; Rawat et al., 2021). Acidification, chelation, enzymatic hydrolysis and hyphal nutrient acquisition are all interrelated and are linked to mineral mobilization in the rhizosphere and enhanced plant nutrition.

#### 4.1.2 Siderophore production

Fungi produce siderophores that form high-affinity complexes with Fe, which is another way that the micronutrients are acquired (Rawat et al., 2021). The acquisition of Fe by siderophores has been reported for *Gigaspora margarita*, *Aspergillus*, and *Saccharomyces* (Rajapitamahuni et al., 2023). This process is especially important under salinity conditions, as limited Fe availability can hinder chlorophyll biosynthesis and photosynthesis (Kabir et al., 2020). Better Fe acquisition may also contribute to antioxidant and electron-transfer functions dependent on Fe, thus connecting micronutrient nutrition to oxidative-stress protection.

#### 4.1.3 Homeostasis of ions and osmotic adjustment

High levels of Na^+^ and Cl^–^ impair the assimilation of nutrients and ionic homeostasis of cells (Evelin et al., 2019). Fungal colonization can help to limit Na+ accumulation and increase K^+^ and Ca^2+^ uptake, which helps to maintain favorable K^+^/Na^+^ and Ca^2+^/Na^+^ ratios. In the case of *Robinia pseudoacacia*, AMF inhibited the uptake of Na^+^ into the xylem and facilitated the movement of K^+^ toward the shoots (Chen et al., 2017). Fungal-induced salinity tolerance has been correlated with the regulation of Na^+^/H^+^ antiporters, K^+^ transporters, proton pumps and aquaporins for ion compartmentation and water transport at the molecular level. Fungi also affect compatible solutes like proline, glycine betaine and soluble sugars which are involved in osmotic adjustment (Sagar et al., 2021; Ondrasek et al., 2022). Lower accumulation of proline in some plants successfully colonized, however, may be due to lower stress level, not less osmoprotection.

#### 4.1.4 Antioxidant and hormonal regulation

Fungal colonization reduces the oxidative damage caused by salinity by controlling systems that scavenge ROS. For instance, *Serendipita indica* (*Piriformospora indica*) increases the activity of SOD, CAT, POD, APX and GR and decreases the accumulation of H_2_O_2_ and malondialdehyde (Boorboori and Zhang, 2022). These responses serve to maintain the integrity of cellular membranes and nutrient transport systems, and are associated with redox regulation and mineral homeostasis. Fungi also alter the hormonal signal transduction pathways of ABA, GA, IAA, ethylene, cytokinins and brassinosteroids (Ryu and Cho, 2015). Auxin levels in plants can be modified by endophytic fungi, including *Phoma herbarum*, *Penicillium* sp., and *Trichoderma asperellum* (Gupta et al., 2021), and auxin responses induced by fungi stimulate lateral-root formation (Contreras-Cornejo et al., 2009). Accordingly, AMF colonization can increase root length, surface area and biomass (Harris-Valle et al., 2018), thereby increasing the soil volume explored for water and nutrients.

### 4.2 Plant-fungal interactions for mineral solubilization and stress tolerance

Salinity interaction between fungi and plants is a two-way process between rhizosphere processes and host metabolism. Root exudates supply nutrients and signaling molecules that affect fungal recruitment and activity, whereas fungi alter nutrient availability, root architecture and plant stress responses (Yusuf et al., 2025). In symbiotic associations fungi provide mineral nutrients, especially P and N, and plant-derived carbohydrates and lipids (Campos, 2020).

Improved salinity tolerance has been reported in plants associated with beneficial fungi, including *Eleusine coracana* (Mahadik and Satyan, 2025), *Solanum lycopersicum* (Hashem et al., 2015), *Stevia rebaudiana* (Bahari Saravi et al., 2022), and *Triticum aestivum* (Khan et al., 2022). While there are some differences in specific species of fungi and plants, the following are some of the most commonly reported improvements in mineral nutrition, ion balance, photosynthetic performance, antioxidant capacity, and hormonal regulation. An example of functional complementarity is the increase in P pool by PSF and the acquisition and transfer of nutrients by AMF hyphae to the host (Etesami et al., 2021). Complementary interactions between N-, P-, and K-mobilizing fungi were observed under salinity, and similar interactions with K-, Zn-, and Fe-mobilizing fungi are worth investigating.

Under salinity, the growth of tomato is enhanced by *Serendipita indica* (*Piriformospora indica*) and the changes in the root branching, biomass, chlorophyll, antioxidant enzymes and IAA associated responses (Fontana et al., 2021). On the other hand, lower levels of proline and ABA were found in colonized plants (Abdelaziz et al., 2019), indicating that fungal inoculation could either trigger protective mechanisms or decrease the degree of stress. In a similar way, *Trichoderma harzianum* and *T. reesei* enhanced salinity tolerance in finger millet by altering the levels of proline, IAA, salicylic acid, chlorophyll, and electrolyte leakage (Mahadik and Satyan, 2025). These observations collectively suggest that the groups of fungi share similar processes for mineral acquisition, ion homeostasis, root growth, redox regulation and hormonal signaling but do not use a single universal mechanism. The positive effects of fungal inoculation on plant growth, nutrient acquisition, photosynthetic potential, and biochemical and physiological functioning can contribute to enhanced plant tolerance to salinity (Borde et al., 2017).

Due to the different nature of the endpoints, the available evidence should be considered in the context of experimental scale–fungal mineral solubilization activity and salinity tolerance of plants are not synonyms. The ability of a fungal isolate to solubilize mineral nutrients, produce organic acids, siderophores and/or extracellular enzymes, and tolerate defined salt concentrations can be determined *in vitro*, but this does not necessarily equate with the ability of the fungus to provide nutrients to plants in soil. Evidence from greenhouse and growth-chamber experiments is more definitive as it includes host responses, root colonization, uptake of nutrients and ion homeostasis under controlled salinity. Agronomic relevance is highest with field experiments, but these are also subject to more variability due to soil properties, host genotype, indigenous microbial communities, climate and management practices. The studies summarized in Table 4 are therefore regarded as being part of an evidence continuum and not as directly comparable, demonstrations of fungal efficacy.

**TABLE 4:** Experimental evidence for fungal-mediated mineral mobilization and salinity stress mitigation in plants.

S. no.	Plant	Fungi	Experimental system	Salinity regime	Mineral/nutritional focus	Major effects	Major limitations	References
1	*Oryza sativa* (Rice)	*Acaulospora mellea*, *G. formosanum*, *Rhizophagus clarus*, *Glomus* sp.	Greenhouse; sand culture	200 mM NaCl	P; K^+^/Na^+^ balance	Improved biomass, P uptake, photosynthesis and ion balance	Controlled sand culture; limited field relevance	da Silva et al., 2022
2	*Hordeum vulgare* (Barley)	*Glomus* sp., *Gigaspora* sp.	Field; saline soil	Saline soil	N, P, K	Increased nutrient uptake, growth and grain yield	Response influenced by P fertilization and field conditions	Masrahi et al., 2023
3	*Triticum aestivum* (Wheat)	*Aspergillus niger*, *Trichoderma harzianum*	*In vitro* + plant assay	60 mM NaCl	P; phytase/ phosphatase	P solubilization and improved wheat growth; IAA and enzymes produced	Controlled assay; no field validation	Gaind, 2016
4	*T. aestivum* (Wheat), *S. bicolor* (Sorghum), *B. juncea* (Mustard), *P. glaucum* (Pearl Millet)	*Penicillium*, *Aspergillus* spp.	*In vitro* + seed assay	1%–5% (w/v) NaCl	P; siderophores; IAA	Salt-tolerant P solubilization; improved germination and seedling growth	Mainly *in vitro*/early-stage evidence	Chandra et al., 2024
5	*Solanum lycopersicum* (Tomato)	*R. irregularis*	Controlled plant experiment	100 mM NaCl	K^+^/Na^+^; hormones	Improved K^+^/Na^+^ balance, growth and hormonal regulation	Controlled experiment; GA3 interaction	Khalloufi et al., 2017
6	*Pisum sativum* (Pea)	*R. intraradices*, *Funneliformis mosseae (G. mosseae)*, *Rhizophagus fasciculatus*, *Gigaspora* sp.	Plant experiment	Salt stress	Nutrients; ions; antioxidants	Improved growth, nutrient uptake, antioxidants and ionic balance, enhanced yield by 11%–54%	Controlled conditions; fungal responses varied	Parihar et al., 2020
7	*Zea mays* (Maize)	*Funneliformis mosseae (G. mosseae)*, *Candida membranifaciens*	Controlled plant experiment	NaCl stress	P; siderophores; IAA	Improved biomass, photosynthesis, antioxidants and P solubilization	Different fungal experimental designs	Sheng et al., 2011; Jan et al., 2022
8	*Aeluropus littoralis* (Grass)	*Claroideglomus etunicatum*	Greenhouse; sand culture	50, 200 mM NaCl	K^+^/Na^+^; N; water relations	Increased biomass, K^+^/Na^+^ ratio, photosynthesis and water-use efficiency	Halophytic host; sand-culture system	Hajiboland et al., 2015
9	*Citrullus lanatus* (Watermelon)	*Funneliformis mosseae (G. mosseae)*	Greenhouse; pot experiment	Salinity–alkalinity stress	N, P, K, Ca, Mg, Fe, Zn	Improved nutrient uptake, photosynthesis, antioxidants and stress responses	Combined salinity–alkalinity stress	Ye et al., 2019

### 4.3 Critical comparison of evidence across laboratory, greenhouse and field studies

Fungal-mediated mineral solubilization and salinity tolerance have been demonstrated in laboratory, greenhouse, and field experiments, but their experimental controls and ecological relevance vary. Laboratory assays can be used for screening of functional traits like phosphate solubilization, production of organic acids, siderophores, extracellular enzymes and tolerance to NaCl. For instance, the halophilic fungi obtained from salt-contaminated soils have been found to possess phosphate-solubilizing activity under saline conditions, which could make them good bioinoculants. Nevertheless, *in vitro* activities do not always correlate with the capacity to promote plant growth and mobilizing nutrients in soil, as the activity of fungi may be influenced by the composition of the substrate, its pH, and its interaction with microorganisms and plant-derived signals (Chandra et al., 2024).

Responses to salinity at the plant level, such as root colonization, uptake of nutrients, biomass, ion balance, photosynthesis, and antioxidant responses are more easily investigated in greenhouse and growth-chamber studies. *Piriformospora indica* for instance enhanced root branching and biomass and modified the antioxidant and Na^+^/K^+^ responses in tomato plants subjected to 200 mM NaCl (Abdelaziz et al., 2019). Further, a meta-analysis of 97 studies showed that AMF tended to generally enhance shoot and root biomass under saline conditions, but this response depended on the different groups of AMF fungi, host plant properties, and salinity level and treatment time. However, responses to controlled substrates and consistent salt exposure and minimal competition by microbes may be more reproducible than those of agricultural production systems.

Field studies are more realistic in terms of agronomic relevance, but tend to have higher response variability. In saline wheat soil (EC 7.41 dS m^–1^), it was found that AMF inoculation resulted in higher growth and nutrient uptake, but the responses varied significantly among AMF species and wheat genotypes, indicating host–fungus compatibility (Daei et al., 2009). Likewise, a meta-analysis of 38 field trials revealed that AMF had beneficial effects on wheat biomass and grain yield, nutrient acquisition, soil organic matter, soil pH, soil nutrient availability, soil texture, climate, and AMF identity (Pellegrino et al., 2015).

The difference between controlled and field evidence also is apparent at the yield level. A meta-analysis of 70 field inoculation experiments gave an average of 16% increase in cereal grain yield, although only 77% of the experiments resulted in a positive response, with neutral and negative responses occurring as well. The answer was different depending on the crop and cultivar, and soil pH (Zhang et al., 2019). Therefore, laboratory and greenhouse experiments can be used to reveal mechanisms and the identification of potential fungal strains, while field testing is required to demonstrate repeatability and field applicability of these effects.

Overall, the evidence suggests a sequential validation strategy: Fungal functional potential determined in the lab, controlled stress plant–fungus interactions in the greenhouse, and environmental robustness and yield benefits tested in field trials. It should therefore not be assumed that a positive result at one experimental scale would necessarily be the same at another. More specifically, *in-vitro* mineral solubilizing activity does not necessarily mean the same in biomass production under greenhouse salinity conditions and under field conditions. This variability is further compounded by variation in fungal establishment, root colonization and host genotype x indigenous soil microbiota interactions.

### 4.4 Molecular and multi-omics insights

Physiological responses are supported by recent genomic, transcriptomic and metabolomic studies. Under salinity, fungal colonization results in changes in expression of genes involved in ion transport, Na^+^ compartmentation, aquaporins, ROS detoxification, cell-wall modification, hormone signaling, and primary and secondary metabolism. Transcriptomic studies of AMF-infected plants have especially been associated with differential expression of genes involved in ion-transport and antioxidant activity, which is related to the K^+^/Na^+^ balance. The conclusions are consistent with a model in which the fungal colonization process regulates the acquisition of nutrients and cellular ion and redox regulation, but does not directly change the individual stress responses.

Metabolomic analysis also shows alterations in organic acids, amino acids, soluble sugars, phenolics and other stress-related metabolites. Organic acids are particularly important as they play a role in mineral mobilization in the rhizosphere and also in carbon and redox metabolism in the cells. The presence of changes in organic acid profiles does not, however, indicate that the organic acids themselves led to mobilization of minerals or to an enhanced acquisition of plant nutrients. This can be best achieved by establishing a fungal linkage between changes in rhizosphere chemical speciation and changes in soluble nutrient pools, and ultimately plant nutrient flux or isotope-derived nutrient acquisition. The integrated analysis of plant and fungal transcriptomes, combined with metabolomics and ionomics of the rhizosphere is still limited. These methods, with functional validation and isotope tracing, are required to differentiate between causal and correlation mechanisms and to estimate the contributions of fungi to the acquisition of P, K, Zn, Fe and Ca under salinity conditions.

### 4.5 Field-level constraints and future perspectives

The capability of beneficial fungi to enhance mineral availability, nutrient uptake, ion homeostasis and plant performance under salinity has consistently been established both under laboratory and greenhouse conditions; however, the effectiveness of these fungi under field conditions is widely variable. Fungal population and genotype, indigenous microbial population, soil pH and texture, nutrient availability, salinity intensity, salinity type, climate, irrigation, and agricultural management affect field performance. In addition, resident microorganisms compete with introduced fungi and high salinity and other environmental factors may help to prevent establishment and root colonization by fungi. Therefore, the efficacy of fungi should not be regarded as an intrinsic property of the inoculant, but as a context-dependent phenomenon (Zhang et al., 2019; Li et al., 2025).

One drawback is the disconnect between *in vitro* or controlled environment functional potential and reproducible field performance, not only due to the ecological context, but also as a result of factors related to the biological quality, formulation and stability of the inoculum, as well as conditions of storage and application. Variability in propagule viability, inoculum formulation, storage and methods of application, colonization efficiency and environmental conditions may all be factors in inconsistent response. The mineral-solubilizing activity in the laboratory, therefore, should be used as a screening test and not as a direct indicator of nutrient-use efficiency in the field. The uniformity of reporting of fungal identity, inoculum quality, propagule density, colonization, soil characteristics, salinity status and agronomic management would increase the comparability and reproducibility of studies.

In addition to ecological constraints at the field level, the use of fungal inoculants is hindered by limited inoculum quality, formulation, stability and scalability. The commercial formulations need to guarantee the viability and infectiveness of the propagules throughout the production, storage period and transport and the carrier materials should ensure the protection of propagules from drying and environmental changes without affecting the fungal activity. Fungal identity, propagule viability or infectivity, purity, contamination status, formulation consistency, and batch-to-batch performance should be a part of the quality control. The requirements are especially difficult for AMF since they are obligate biotrophic fungi and produce not as easily as fungi like *Trichoderma*, *Aspergillus* and *Penicillium*. Therefore, development of the formulation should include stability and shelf-life testing under predetermined storage conditions instead of just initial propagule concentration (Basiru and Hijri, 2022; Salomon et al., 2022; Ghorui et al., 2025).

Optimization of production cost, carrier compatibility, application method, field persistence and regulatory quality standards are additional requirements for commercial scalability. A formulation that is effective in the greenhouse can be ineffective after storage, transport or application due to loss of propagule viability and some environmental heterogeneity and competition with indigenous microorganisms. Thus, commercialization should be done in a step-wise manner including strain authentication, scalable production, formulation and shelf-life validation, standardized quality control, multi-location field testing and economic feasibility. Compatibility and functional stability in production and storage should also be tested for fungal consortia. This could be useful to close the gap between proven fungal activity and commercially viable, repeatable and reproducible inoculants in saline and nutrient-limited soils.

There are also gaps in knowledge regarding host–fungus specificity and/or colonization stability. Root exudation, root architecture, carbon allocation and root signaling are host genotype dependent, while fungal isolates vary in their nutrient mobilizing ability and stress tolerance and in their root colonization behavior. Therefore, the efficacy of fungi should be assessed in the context of host × fungal genotype, rather than the fungal species alone. Likewise, the presence of fungal structures or DNA in the soil does not necessarily mean that the fungi are established or active in the soil. An introduced fungus could be a competitor of native microorganisms and react differently to the environmental changes that occur throughout the year. Functional indicators like nutrient transfer, mineral solubilization, ion balance and yield (Basiru and Hijri, 2022) should, therefore, be associated with repeated evaluation of colonization throughout the crop cycles and successive seasons when applicable.

It is therefore necessary to validate the fungal-mediated benefits in long-term multi-season, multi-location field experiments to establish their stability under contrasting soils, salinity levels and climatic conditions. These should be used to test biomass production and yield as well as nutrient-use efficiency, tissue Na^+^/K^+^ balance, root colonization, soil nutrient availability and interactions with indigenous microbial communities. Field-testing strategies should include genotype × environment × management interactions to discover locally adapted strains or combinations of fungi with predictable benefits under particular agroecological conditions.

Further studies should also proceed from testing single isolates to functionally tested fungal consortia such as AMF in combination with P, K, Zn or Fe-mobilizing fungi. However, compatibility, persistence and functional stability should not be taken for granted, and the potential synergistic effects should be experimentally demonstrated. A staged validation approach (from strain screening, host compatibility tests to controlled studies, replicated field tests and multi-location, multi-season tests) would yield more field applicability evidence. Combining the effects of fungal colonization, mobilization of minerals, nutrient acquisition, ion balance, and physiological reactions and yield would clarify mechanistic effects from measurable agronomic gains.

Fungal-mediated salinity tolerance is an integration of rhizosphere mineral mobilization and plant ion, redox, osmotic and hormonal regulation. The ability to increase nutrient availability through the use of organic acids, extracellular enzymes, and siderophores, as well as by modifying fungi hyphae and roots for nutrient uptake and stress control, can be beneficial. While this holistic model is supported by an increasing number of molecular and multi-omics studies, there remains a lack of field-scale mechanistic validation and persistence/reproducibility studies. So, evidence for fungal-mediated mineral solubilization and salinity tolerance should be considered a continuum, from laboratory and greenhouse studies that demonstrate functional potential to ecological stability and agronomic reliability in long-term, multi-location field trials (Table 5).

**TABLE 5:** Study type for evaluating applicability and limitations of laboratory, greenhouse and field studies for fungal-mediated mineral solubilization and salinity tolerance.

Study type	What the study type can establish	Key limitations of the study type
Laboratory / *in vitro* evidence	• Fungal tolerance to defined salinity levels • P, K, Zn and Fe solubilization potential • Organic-acid and siderophore production • Extracellular enzyme activity • Identification and screening of promising fungal strains	• Does not reliably demonstrate plant nutrient acquisition • Does not establish stable root colonization under soil conditions • Cannot reliably predict biomass or yield benefits • Does not demonstrate persistence or effectiveness under field conditions • Simplified culture conditions may not reflect soil–plant–microbial interactions
Greenhouse / growth-chamber evidence	• Host–fungus compatibility • Root colonization and establishment under controlled conditions • Plant biomass and nutrient uptake • K^+^/Na^+^ balance and ion homeostasis • Antioxidant and osmotic responses • Physiological and molecular mechanisms of salinity tolerance	• Often uses simplified substrates or controlled soil conditions • Salinity intensity and exposure are usually artificially regulated • Indigenous microbial competition may be limited • Temperature, water availability and other environmental variables are controlled • Short experimental duration may not capture long-term persistence or yield stability • Positive responses may not translate directly to field conditions
Field evidence	• Agronomic reproducibility under realistic conditions • Fungal persistence and establishment in agricultural soils • Interactions with indigenous microbial communities • Crop growth and yield responses • Environmental robustness across soil and climatic conditions • Practical feasibility of fungal inoculation	• High environmental and soil heterogeneity • Colonization and inoculum performance may be inconsistent • Strong site-, crop- and genotype-specific responses • Formulation, storage and application methods can affect efficacy • Climatic and management variability complicates comparisons • Economic feasibility and long-term persistence require further validation
Formulation and commercial validation	Propagule viability, stability, shelf life, carrier compatibility, batch consistency, application efficiency	Production cost, contamination, storage losses, regulatory requirements and scalability

## 5 Genetic insights into plant-fungal interactions under stress and nutrient deficiency

The molecular tools have greatly enhanced understanding of the role of beneficial fungi in regulating plant salinity and nutrient limitations. Sequencing, genomics, transcriptomics, proteomics and metabolomics can be used to identify microbial functional traits, as well as the molecular processes involved in plant–fungal interactions (Dabban et al., 2024). These datasets can also be integrated using bioinformatics to predict microbial ecological functions and the potential to reduce stress (Morales-Vargas et al., 2024). Importantly, fungal-mediated stress tolerance is not controlled by individual genes, but by networks of genes that regulate ion homeostasis, redox balance, nutrient acquisition, hormonal signaling, root development and fungal colonization.

### 5.1 Distinct molecular responses of fungi and plants under salinity and nutrient limitation

#### 5.1.1 Fungal molecular responses

Several genes in plants and fungi have been identified that are related to stress adaptation and nutrient mobilization (Behie and Bidochka, 2014; Porcel et al., 2012). In response to salinity, fungi re-program their metabolism and express stress-related genes which play a role in protein stability, cell integrity and halotolerance. In *Penicilliopsis clavariiformis*, there was a correlation between salinity and changes in the expression of stress-related genes such as Hsp98, Hsp60, HTB and RHO (Kashyap et al., 2015). The observations suggest that the heat-shock, chromatin-associated and cellular-regulation pathways are transcriptionally involved in the adaptation of fungi to saline conditions. It should be noted that differential expression does not imply a causal relationship between any particular gene and fungal halotolerance, and further functional validation would be needed to conclude such a relationship for a particular gene under stressful conditions.

#### 5.1.2 Plant molecular responses induced or modulated by beneficial fungi

Fungal colonization can then alter plant pathways related to antioxidant defense, ion transport, and hormonal signaling and nutrient uptake (Verma et al., 2021). ROS build-up under salinity stress damages lipids, proteins and nucleic acids (Hasanuzzaman et al., 2021). Plants cope with this stress by means of antioxidant systems of CAT, APX, SOD, and GPX (Aleem et al., 2022). Under NaCl condition, the activities of antioxidant enzymes and the expression levels of SOD, POD and CAT gene in *T. longibrachiatum* inoculated wheat plants were higher than uninoculated wheat plants (Zhang et al., 2016). Therefore, the involvement of each of the antioxidant genes in the salt-tolerance phenotype of the fungus cannot be ruled out, and the current study offers no proof that any individual gene is essential for the phenotype.

Modulation of the pathways involved in ion-homeostasis within plants is another critical aspect of fungal-associated salinity tolerance. In a number of plant–fungus associations, fungal colonization is linked to changes in the expression of genes involved in the cation transport systems such as NHX-, SOS1-, and HKT-related systems. These transporters are known to play important roles in plant Na^+^ and K^+^ homeostasis: NHX proteins are involved in the sequestration of Na^+^ and K^+^ in the vacuole; SOS1 is involved in the efflux of Na^+^ from the plasma-membrane; and HKT proteins are involved in the transport and/or retrieval of Na^+^ and K^+^ in the plasma-membrane and, in some species, in the xylem. But the biochemical or physiological roles of these transporters should be differentiated from evidence from plants inoculated with fungi alone. For instance, AM symbiosis in rice was correlated with the modulation of the expression of genes involved in cation-transport and resistance to salinity which resulted in reduced Na^+^ transport from roots to shoots (Porcel et al., 2016). Likewise, under salt stress, the presence of *S. indica* (*P. indica*) led to higher transcript levels of LeNHX1 and changes in Na^+^/K^+^ homeostasis (Abdelaziz et al., 2019). While these observations suggest a link between fungal colonization, transporter gene expression and enhanced ion homeostasis, they cannot provide evidence for a direct effect of AMF or *S. indica* (*P. indica*) on transporter activity nor evidence for the requirement of any particular transporter for the observed salt-tolerance phenotype. Functional attribution would require complementary evidence such as protein-level measurements, transport assays, genetic perturbation, complementation or other mechanistic validation.

#### 5.1.3 Evidence for gene function vs. expression association

In AM symbioses, fungal phosphate transporters are crucial for the acquisition of nutrients; however, the functional evidence is different for each transporter. In *Rhizophagus irregularis*, the expression of GintPT is affected by the symbiotic status and phosphate availability, suggesting an involvement of GintPT in fungal phosphate acquisition, but not causing it (Maldonado-Mendoza et al., 2001; Fiorilli et al., 2013). On the contrary, with the heterologous expression system and gene-silencing studies, functional expression of Pi transport and role in arbuscule development and symbiotic Pi delivery were observed in RiPT7 (Xie et al., 2022). Therefore, it is best to use GintPT as an expression or association level measure and RiPT7 as a functionally validated measure of a phosphate transporter.

The fungal nutrient acquisition machinery is supplemented by the plant nutrient transport systems. Beneficial fungi influence the plant PHT family transporters and facilitate the uptake of nutrients from the symbiotic interface. For example, *Penicillium olsonii* colonization was associated with enhanced phosphate uptake and altered expression of PHT1- and PHO1-related pathways, together with changes in genes associated with root development and hormonal signaling (Agisha et al., 2024). These findings support coordinated changes in phosphate acquisition and root-development pathways, but do not establish that any individual transporter or signaling gene is solely responsible for the phenotype. Therefore, Figure 2 reflects a cooperative interaction; fungal responses, in form of stress tolerance and mobilizing nutritional resources, coupled with plant processes including ion transport, defense mechanism against oxidative damage, hormone-mediated processes and nutrient uptake.

**FIGURE 2:**
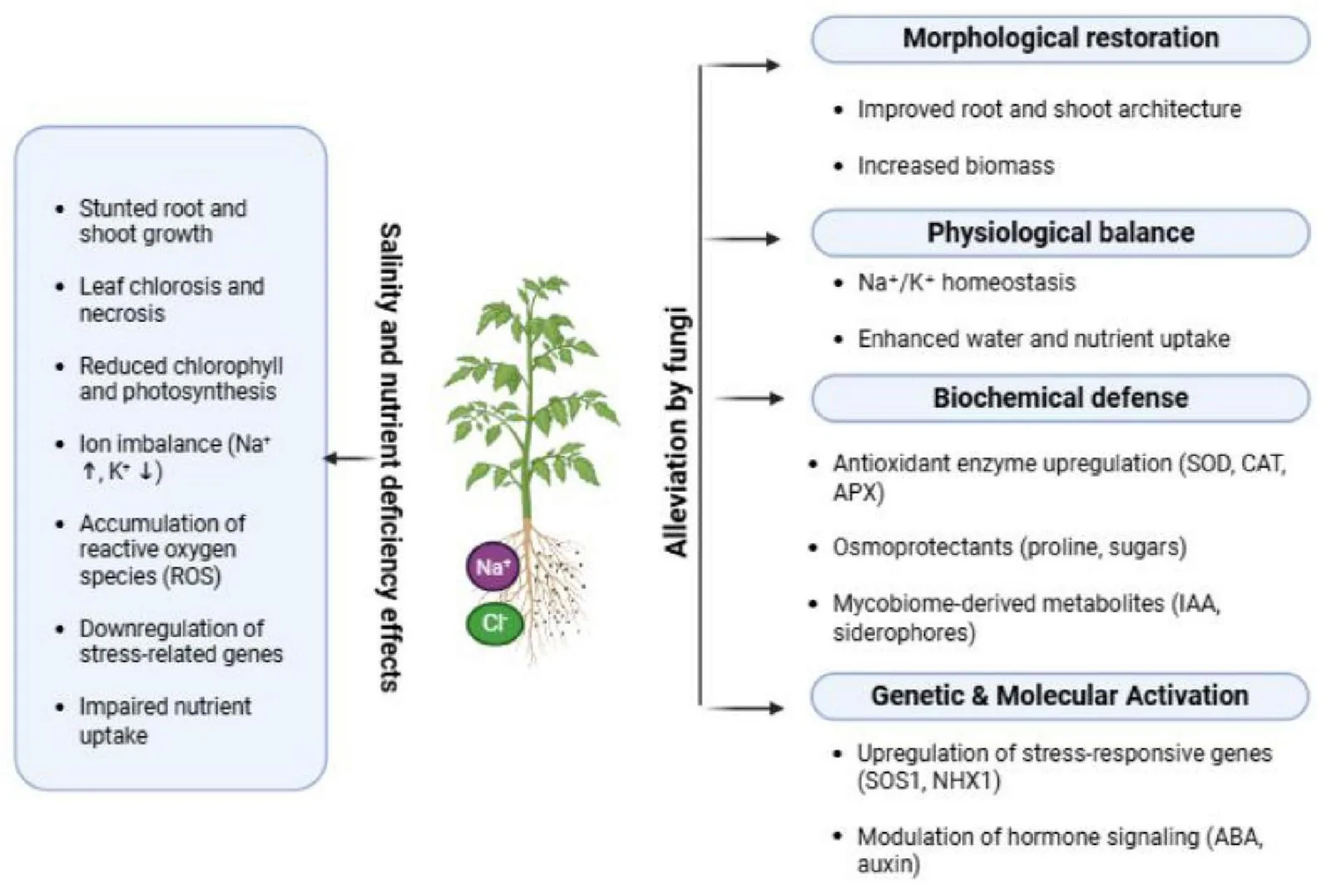
Fungal-mediated alleviation of salinity and nutrient deficiency stress in plants. Salinity stress causes ion imbalance, oxidative damage, nutrient deficiency, and impaired plant growth. Beneficial fungi mitigate these adverse effects through morphological, physiological, biochemical, and molecular mechanisms, including ion homeostasis, antioxidant defense activation, nutrient solubilization, hormonal regulation, and stress-responsive gene expression, which are associated with enhanced plant growth and stress tolerance in several experimental systems.

### 5.2 Insights from omics technologies

Genomic analysis identifies genes and pathways known to play functional roles in fungi or plants, while transcriptomics measures changes in RNA abundance under specific environmental or symbiotic conditions. Proteomic analysis can provide evidence on changes in protein abundance, although protein abundance does not necessarily correspond to enzymatic or transport activity. Metabolomic and ionomic analyses can provide information on downstream phenotypes, namely biochemical and elemental phenotypes. Consequently, differential expression of a gene should not be used as evidence of functional activity of the protein or as evidence that the gene is directly responsible for a physiological phenotype. The biochemical assays, nutrient/ion measurements, protein abundance, heterologous expression, gene silencing, mutant phenotype and complementation studies all give increasing support for the functional interpretation. The strength of inference should therefore be matched to the experimental evidence available.

Omics technologies helped to shift the plant and fungi interactions from a single gene study to a system-wide study of adaptation to stresses (Figure 3). Integration of molecular and physiological data through comparative approaches has identified the systemic alteration in gene expression, protein activity, metabolism, ion transport, and hormone signaling networks during salinity stress (Ullah et al., 2022; Arif et al., 2020). Recent studies further reinforce that endophyte-facilitated stress tolerance is a multifactorial process mediated by a complex network of molecular, metabolic and signaling pathways which has been better elucidated by multi-omics approaches (Mohammed et al., 2026).

**FIGURE 3:**
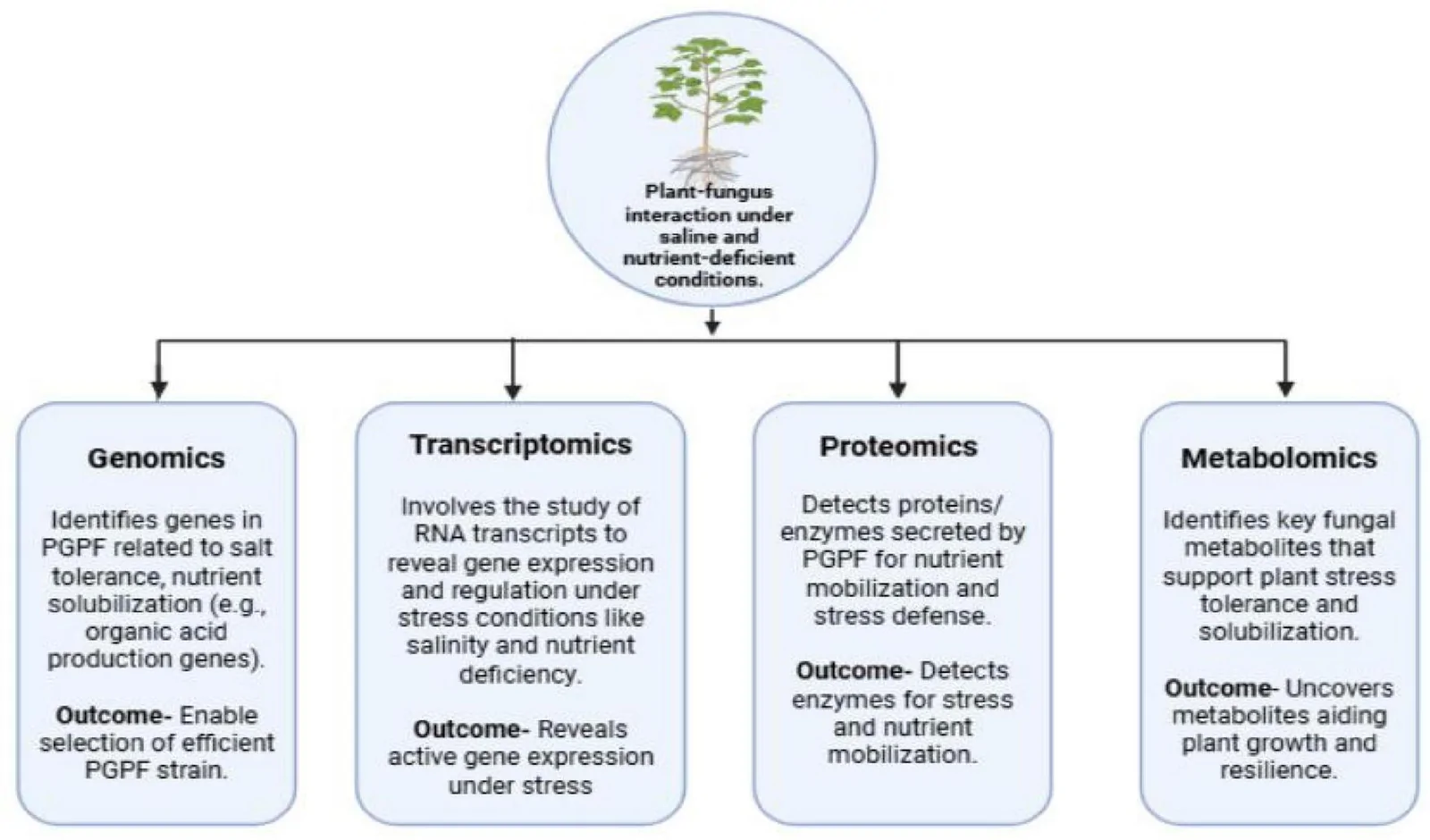
Omics-based analysis of plant-fungus interactions under saline and nutrient-deficient stress.

Genomics provides insight into the beneficial functional potential of fungi by identifying genes associated with nutrient metabolism, stress responses, transport, secondary metabolism, and plant interactions. Accordingly, genome sequencing approaches, coupled with comparative genomics, have broadened the scope of functional analyses of plant-associated microorganisms (Abdullah et al., 2024). For instance, *Cladosporium* sp. has genes encoding enzymes for the biosynthesis of tryptophan, sterols, and brassinosteroids, as well as genes involved in nitrogen metabolism and stress response, indicating a wide array of functional responses to stress-associated factors that contribute to plant development (Yang et al., 2023). However, the present genomic evidence reflects the functional potential rather than functional implementation/operation.

Transcriptomics offers a dynamic overview of genes whose transcript abundance changes during plant-fungal interaction (Rajawat, 2018). For example, *Trichoderma* has been found to modulate photosynthesis, root architecture and plant defense at the transcriptomic level (Abdullah et al., 2024), although genes related to stress-, metabolic-, and transport functions were identified as differentially expressed between *S. indica* (*P. indica*)-treated and non-treated date palm in response to salinity (Ahmad et al., 2024). Dual RNA-seq and spatial transcriptomics add further dimensions to understanding these plant-fungus responses, enabling discrimination of transcript profiles in host and fungus along the site of colonization.

Proteomics complements transcriptomics because expression levels do not necessarily correlate with functional protein levels. For example, the proteome data on *Trichoderma* (Leetanasaksakul et al., 2024) associated various proteins related to stress tolerance, cell signaling, nutrient metabolism, and energy production with symbiotic processes, while similar observations revealed forty-six proteins relevant to stress defense, signaling, metabolism, and energy production were involved in the response to the *S. indica* (*P. indica)* colonization in *Brassica napus* under stress (Lv et al., 2014). Integrating transcriptomic data with protein profiling is, therefore, necessary to distinguish expression from functional aspects of gene responses.

The metabolome encompasses metabolites affected in the aftermath of biochemical changes and reflects the contribution to stress adaptation from compounds such as osmolytes, organic acids, antioxidants, phytohormones, and other secondary metabolites (Wu et al., 2023; Ali et al., 2024). *Trichoderma atroviride* induced modulation of Arabidopsis thaliana root architecture by means of production of 6-pentyl-2H-pyran-2-one(6-PP) which alters levels of signaling molecules such as auxin and ethylene (Garnica-Vergara et al., 2015), demonstrating how plant–fungus interactions can influence plant traits via the secretion of chemical signals in addition to gene expression patterns.

Integration of an orthology database (e.g., COG/eggNOG) and KEGG pathway analysis identifies the potential involvement of genes in functional metabolic pathways; networks provide information about co-expressed modules of genes involved in the regulation of the nutrient balance, ion homeostasis, osmotic pressure, and stress tolerance (Sefrji et al., 2025). The approaches outlined in Figure 3 are complementary, with the omics-based approach encompassing Genomics (identifying potential), Transcriptomics (evaluating temporal and conditionality dependent gene expression), Proteomics (approximating real functioning and level of expressed protein product) and Metabolomics (approximating conditionality dependent effect resulting from functional expression of genes).

### 5.3 Research gaps in current omics-based studies

Although the current studies have achieved significant insights, many aspects of the molecular mechanism underlying the adaptation to salt and low nutrient conditions is not thoroughly understood. Firstly, existing knowledge remains focused on plant functions and fewer studies have investigated fungal genes, which are important in nutrient solubilization and provision, metabolism and transport of nutrients, maintenance of cellular homeostasis, colony expansion and nutrient transfer between fungus and plant. Secondly, salt stress and nutritional shortage are often investigated individually, whereas these conditions frequently co-exist, their interactive effects remain insufficiently characterized. Most Studies examine whole tissue, only a few are conducted at a certain time point, that might result in omitting cell specificity and transient responses. Furthermore, differential expression of genes or abundance of proteins and metabolites generally indicates association rather than causation. One of the key limitations of present molecular investigations is that changes in expression, protein abundance or metabolite accumulation are, in the majority of cases, only association and not causation. Functional attribution needs perturbation of the candidate gene/pathway, followed by the assessment of the molecular and physiological phenotype. Thus, candidate genes identified by transcriptomic or genomic approaches should be regarded as potential regulators until confirmed by loss-of-function, gain-of-function, complementation, heterologous expression, or biochemical or transport assays. Accordingly, throughout this review, terms such as “associated with,” “correlated with,” “consistent with,” and “suggests” are used for expression-level observations, whereas stronger terms such as “demonstrates,” “mediates,” or “is required for” are reserved for studies providing direct functional or genetic evidence.

### 5.4 Limitations of omics approaches and possible solutions

Complexity of biology itself makes certain data difficult to extract, incomplete Annotation of fungus genome, and also challenges distinguishing between plant and fungal signals at different levels and scales are factors hampering data interpretation (Xie et al., 2025). It is even possible that expression levels and corresponding protein concentrations and metabolic activities differ in many respects. Dual-RNA-seq and single-cell or even spatial transcriptomics combined with other methods like targeted quantitative proteomic analysis, spatial proteomic, spatial metabolomics, phosphorylation of protein and high-throughput measurement of osmolytes would be of great help for overcoming these problems (Katam et al., 2022; Ali et al., 2026; Kundu and Tanti, 2026). Quantitative analyses of nutrient flow with stable isotope feeding can provide evidence of direct transfer; and functional validation of the gene set using CRISPR-technology or using gene silencing will permit determining the role of key players. Thus, the integration of various datasets, and network analysis, and machine-learning approaches for data processing would help us overcome these limitations.

### 5.5 Future perspectives: toward integrated multi-omics analysis

In our opinion, future study should concentrate on data integration and prediction models based on existing omics data. Comprehensive approaches are therefore indispensable including plant and fungal genome sequences with data from both host and parasite. Spatial transcriptional profiling, quantitative proteomics and metabolomics studies will be especially beneficial for obtaining holistic information about interactions occurring *in planta* with respect to the development of molecular patterns in both organisms. The resulting information can integrate plant ion, nutrient content, root architecture and growth in different stress conditions. The importance of integration of multi-omics analyses will be essential when aiming to elucidate multi-faceted mechanisms underlying fungal-induced tolerance to salt/low nutrients, since plant tolerance is a result of complex signaling networks regulating the hormonal profile, oxidative stress, nutrients uptake and metabolism (Mohammed et al., 2026).

Also, machine-learning prediction models can help understand complex interactions among all relevant players in the interactions between beneficial fungi and plants when these interactions occur simultaneously under different stress conditions. However, in-silico studies must be complemented with *in vivo* and lab validation and physiological analyses.

The tolerance effect conferred by the beneficial plant-microbe interaction is not generated solely by the active stress-responsive gene/metabolic activity of a relatively few genes, but rather results from complex network interactions among molecular players in both microbes and plant hosts under specific conditions. Integrating omics data at different levels, spatial approaches, physical nutrient flow measurements, functional genomics analyses, and physiology experiments would facilitate the efficient design of novel plant-beneficial fungi for use as inoculants in saline and nutrient-deficient soils.

## 6 Future perspective

Currently, sustainable agriculture that fosters production at a scale that can guarantee human food security without adverse effects on the environment is being encouraged worldwide. Fungi are required to promote healthy plant growth under inhospitable conditions, such as salty and nutrient-deficient conditions. They can also be employed as close companions in sustainable agriculture because of their ability to enhance root architecture, mineral solubilization, and regulation of stress-responsive genes. In addition to enhancing nutrient absorption, arbuscular mycorrhizal fungi and other beneficial soil fungi preserve soil structure and microbial balance. These attributes render fungi potential biofertilizers that can reduce the reliance on chemical additives, promote eco-friendly agro-ecosystems, and enhance the resilience of crops in degraded soil environments.

Further research in this area and enhancement of fungal inoculants into fieldable formulations through the identification of effective fungal strains and development of cost-effective and stable formulations will be essential in the future. The application of these biofertilizers in both conventional and organic agricultural systems can assist in ensuring sustainable food production, particularly in regions with salinity and nutrient shortages. Molecular and genomic technologies can also be used to learn more about plant-fungal interactions and possibly create next-generation biofertilizers that are suitable for specific crops and environmental conditions. We can develop fungal-based solutions that will lead to the development of strong farming systems that can survive in degraded and saline soils without harming the environment.

## 7 Conclusion

This review emphasizes the great potential of fungi as sustainable and environmentally-friendly bio-agents for plant growth promotion under salinity and nutrient-limited conditions. Salinity of soil and mineral deficiencies are major abiotic constraints to crop productivity globally, which affect crop physiology, uptake and productivity. The role of the arbuscular mycorrhizal fungi and endophytes in the alleviation of these stresses can be in several aspects such as mineral solubilization, production of organic acids, enzymatic activity, ion homeostasis and promotion of antioxidant defense system. Plants can improve their tolerance by their ability to modify their root system, phytohormone and ionic balance. In addition, advances in the development of molecular and omics technologies have expanded our understanding of the genetic, transcriptional and biochemical responses associated with stress tolerance and nutrient acquisition, while also revealing the need for functional validation of candidate genes and pathways. The results offer new potential for the creation of specific and effective fungal bioinoculants. Overall, the inclusion of useful fungi in agriculture is a promising approach to reducing the use of chemical inputs, improving soil health, and promoting sustainable crop production. Future research goals should be to identify effective fungal strains and develop economical formulations for effective use in agricultural fields and broader adoption in climate-smart agriculture.
